# Validation of the C.A.R.E. stimulus set of 640 animal pictures: Name agreement and quality ratings

**DOI:** 10.1371/journal.pone.0192906

**Published:** 2018-02-28

**Authors:** Natalie Russo, Carl Erick Hagmann, Rosemary Andrews, Conner Black, Magenta Silberman, Nicole Shea

**Affiliations:** 1 Syracuse University, Department of Psychology, Syracuse, New York, United States of America; 2 Furmam University, Department of Neuroscience, Greenville, South Carolina, United States of America; University of California Berkeley, UNITED STATES

## Abstract

Stimulus sets are valuable tools that can facilitate the work of researchers designing experiments. Images of faces, and line drawings of objects have been developed and validated, however, pictures of animals, that do not contain backgrounds, have not been made available. Here we present image agreement and quality ratings for a set of 640 color images of animals on a transparent background, across 60 different basic categories (e.g. cat, dog, frog, bird), some with few, and others with many exemplars. These images were normed on 302 participants. Image agreement was measured both with respect to the proportion of participants that provided the same name as well as the H-statistic for each image. Image quality was measured both overall, and with respect to the accuracy of participants’ naming of the basic category. Word frequency of each basic and superordinate category based on the English Lexicon Project (Balota, et al., 2007) and the HAL database (Kucera & Francis, 1976) are provided as are Age of Acquisition (Kuperman, Stadthagen-Gonzalez, & Brysbaert, 2012) data.

## Introduction

The development of sets of stimulus images for experimental research has often been a lab by lab endeavor. When these stimulus sets are standardized, they can be a powerful resource for other scientists to use in experimental settings. Stimulus sets of line drawings [1–3], faces [4,5] and objects [6–8] and corpora of images (e.g. SUN database and ImageNet) have proven extremely useful to the scientific community. These have been used for studies that range from examining amygdala responses to medication in depressed patients [9], studies of object categorization in children [10] and adults [11], validations of memory models [12] psycholinguistic studies [13] and studies examining computer vision [14,15] and machine learning [16]. These normed and validated stimulus sets often garner thousands of citations, suggesting that the scientific community values the sharing of stimulus resources.

The stimulus sets that are available are generally comprised of faces, line drawings, or images objects that represent animate human and inanimate categories. Although images, or line drawings of animals are sometimes included in databases, the numbers of exemplars of these are generally very few. In looking through different lists of stimulus databases (e.g. cogsci.nl; cs.cmu.edu), only 3 of more than 40 stimulus databases contained images of animals. Three of these databases contained multiple exemplars of one animal (e.g. 10, 000 cats, 619 butterflies and 600 birds in natural backgrounds), while the other contained approximately 30 images of animals and insects, with only one exemplar of each animal. A database composed only of animal pictures, that contain multiple exemplars across multiple categories can be useful across a variety of experimental settings. In our own research, this database was developed to test the accuracy of temporal visual detection for specific exemplars within categories (e.g. find *this* cat among cats), as well as outside of different types of categories (e.g. find a cat among other four legged animals). This database could be relevant to the study of memory, attention and categorization, and offers many opportunities for developmental studies of various kinds (e.g. across memory, perception, attention and language) as animals are tokens that are often in the vocabulary of very young children. For example, the McArthur Bates Communicative Developmental Inventory [17] for words and gestures, and words and sentences, assess young children’s (between 8 and 18 months of age and 16–30 months, respectively) receptive and expressive vocabulary. Both of these inventories contain many words (400 for the words and gestures, 680 for words and sentences) that children are likely to understand and say, and approximately 10% of these lists represent animal names and sounds, suggesting that they make up a fair portion of typically developing infants and toddler’s vocabulary. Thus, a stimulus set of multiple animal images has broad applicability both across scientific disciplines, but also across developmental ages.

**Fig 1:**
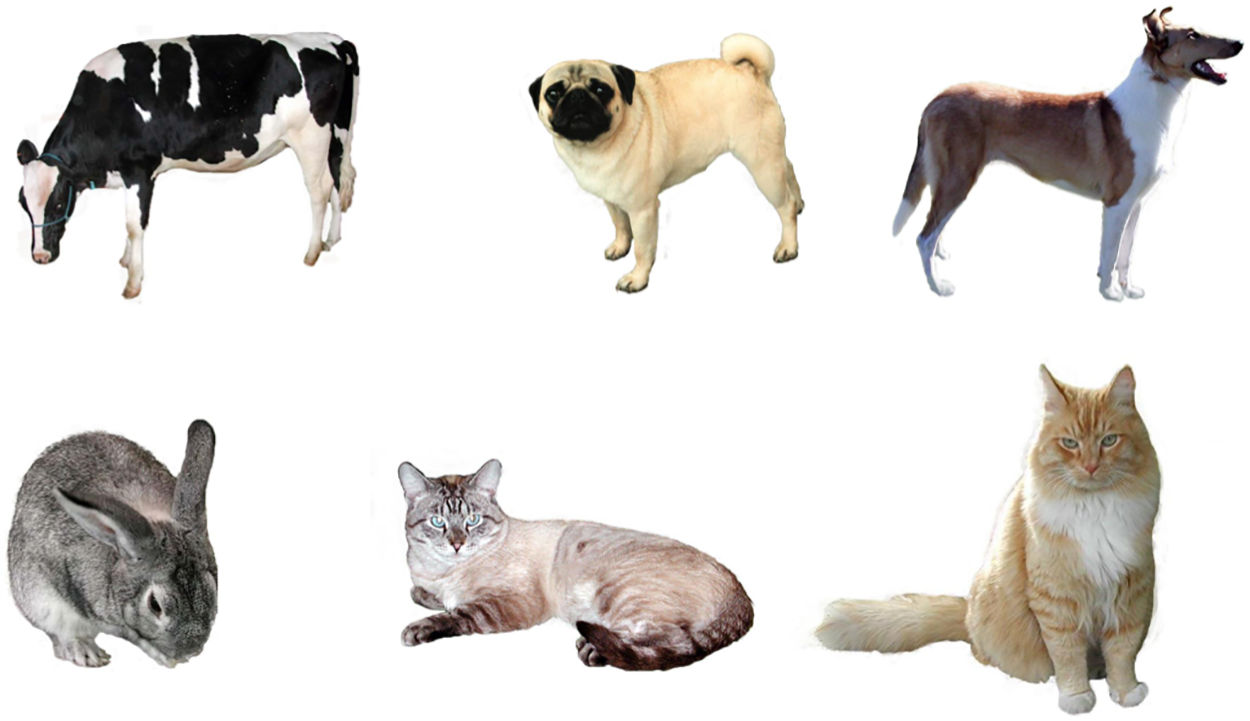
Sample of images from database.

Here we present normative data on a large set of animal pictures (N = 640) across more than 50 basic and eight different superordinate categories. Fig 1 presents a sample of images that were all used and modified with permission. The pictures were culled from multiple databases, photoshopped onto white backgrounds and can be manipulated for size, making them flexible for use in multiple experimental settings.

### Approaches to validation

To validate these stimuli, we used approaches similar to [1,18] and tested a sample of approximately 300 undergraduate participants. Name agreement was defined as the degree to which participants agreed on the name of the pictured animal [1,18]. Name agreement was measured by assessing the proportion of participants who provided the same name for each image, and was completed for both the modal name as well as for the next two most common referents. Name agreement affects naming independently of other attributes including word frequency [19,20]. Further, name agreement is a strong predictor of naming difficulty as images that elicit multiple names are more difficult to identify than those where there is larger naming consensus [1,13,18]. Research has shown that images with high name agreement are identified more quickly and accurately than those with low name agreements [21,22].

In addition to naming agreement we also provide the H-statistic for each image, which is considered a more sensitive measure of name agreement than percentages, providing more information about the name distributions across participants [1]. That is, if participants provide the dominant name for two different images 70% of the time, but in one case the image also elicits 3 other names and in the other case the image only elicits 1 name, then the percent agreements will be equivalent, but the H-statistic will be lower in the former than in the latter. Values close to or at 0 reflect high name agreement, whereas higher values reflect poorer name agreement.

Providing stimuli with both high and low naming agreements offers researchers the opportunity to modulate complexity either between or within tasks using a uniform set of pictures. To further aid in the selection of images appropriate for a variety of research purposes, we also report word frequency for both the basic and subordinate categories via both Kucera & Francis (1976) and HAL database thanks to the English Lexicon Project [23] which can help researchers select either common or infrequent pictures based on their specific research questions. Word frequency has been linked by some to both visual orienting to images [24] and naming speed ([19,21,25] but see [26,27]). Additionally, we also provide age of acquisition (AoA) information, which represent the age at which adults think they learned a word [28]. AoA has been related to the actual ages of acquisition of words and has been shown to correlate highly with lexical decision time [28].

Participants were also asked to rate each image with respect to its fitness as an exemplar of the word they used to describe the image. This provides a metric of the relationship between an image and its mental representation and is intended as a proxy of quality. Images with high ratings correspond to individual’s idea of what a particular stimulus looks like in their mind, which might impact its ease of recognition [21]. We did not ask participants to imagine the animal before rating its representativeness as an exemplar, but rather asked participants whether the image they saw was a good representation of the name they gave that animal. The rating data are presented both overall, that is, irrespective of whether participants correctly identified the basic category, as well as for correct responses.

Finally, we provide the size of each image, as a measure of objective, visual complexity [29], as this has been shown to be correlated with subjective measures of complexity as well as impact picture naming accuracy, while being uncorrelated with RT, word frequency and age of acquisition.

## Materials and methods

### Participants and procedure

302 (197 females) healthy college students participated in the study. Participants were on average 19.4 years old (SD = 3.16), and predominantly right handed (297/302). All participants were over 18 years of age and provided written consent once they had read the description of their study and had their questions answered. The consent form and project had been approved by the ethics review board at Syracuse University. Participants were seated approximately 2 feet away from a Dell computer screen (1920 X 1080 pixel, 60Hz refresh rate), and the task was run on a Mac Mini (2.4GHz). The task was presented via MATLAB [30] and programmed using Stream [31] an interface that uses psychophysics toolbox [32]. Images were resized to be no greater than 500 * 500 pixels and were presented at the center of a screen one at a time in a self-paced manner, with breaks every 25 trials. Each participant viewed 325 distinct images (a subset of the original 650 total images), yielding 150 ratings per image (+/- 2).

Images were presented one at a time, and participants were asked to type the name of the animal (name agreement task) they saw or type ‘don’t know’ or ‘not sure’ (.23% of all observations). They were not directed to the basic, super or subordinate category, and thus, their responses reflect the word they most closely associated with that image. After participants finished typing in their answer they pressed enter and a second screen appeared where they were asked to rate how good of an example the image was of the animal they typed. These ratings were measured on a 7 point Likert scale where 1 represented ‘very bad’, 5 represented ‘good’ and 7 represented ‘very good’. Participants were provided with the anchors for each rating and they remained on the screen until the participant responded. Participants were first presented with a fixation cross, which was replaced 500ms later with a screen asking participants to press enter when ready. The image was then presented until the participant’s first keystroke. If participants did not respond after 10 seconds they were prompted to respond.

### Stimuli

The stimulus pictures were 650 color images (497 horizontal) of animals culled from multiple open access databases, the public domain as well as copyrighted images for which permission to use, modify and distribute was provided. In particular, the Washington State University veterinary image set makes up a large proportion of the images. The images include multiple examples across different basic categories of animals that were familiar (cat, dogs, birds) and a smaller set of examples of images of less familiar animals (e.g. llama, manatee). The numbers of images of each basic category are sorted by superordinate category and are presented in Table 1.

**Table 1:** Number of stimuli per category, overall (strict) percent agreement relative to the basic category, as well as overall ratings, and ratings for accurate responses only, sorted by of superordinate category level.

Superordinate	Basic	N	% agreement	Overall Rating	Accurate Rating
Amphibian	Frog	9	0.86	5.74	5.74
Anthropod	Butterfly	1	0.90	6.47	6.28
Aves	Bird	76	0.37	5.23	5.18
	Duck	16	0.88	5.43	5.64
	Ostrich	2	0.62	5.52	5.85
	Penguin	2	0.79	5.09	5.36
Crustacean	Crab	12	0.62	4.99	4.96
Mammal	Alpaca	13	0.18	5.16	5.33
	Badger	1	0.18	3.30	4.11
	Bear	20	0.54	5.95	5.92
	Camel	1	0.83	5.57	5.76
	Cat	79	0.97	5.87	6.08
	Cheetah	1	0.58	5.09	5.20
	Cougar	1	0.11	5.36	5.94
	Cow	24	0.84	5.07	5.18
	Deer	17	0.86	5.10	5.34
	Dog	141	0.93	5.92	6.02
	Donkey	2	0.78	5.03	5.41
	Eland	1	0.00	3.72	0
	Elephant	5	0.94	6.38	6.53
	Ferret	4	0.27	4.94	5.33
	Fox	8	0.00	4.81	6.50
	Giraffe	1	0.77	6.48	6.65
	Goat	9	0.66	4.29	4.72
	Gorilla	1	0.47	5.43	5.55
	Guinea Pig	7	0.33	5.27	5.70
	Horse	12	0.95	5.56	5.75
	Lion	1	0.81	5.27	5.43
	Llama	5	0.45	5.02	5.25
	Manatee	2	0.37	5.30	5.91
	Mouse	1	0.72	5.16	5.13
	Mule	2	0.04	5.54	5.33
	Okapi	1	0.04	4.76	6.67
	Pig	8	0.78	5.44	5.37
	Prairie Dog	9	0.03	4.33	4.69
	Rabbit	23	0.74	5.70	5.85
	Racoon	1	0.41	5.91	6.23
	Rat	4	0.27	4.31	4.26
	Rhinoceros	10	0.68	5.79	6.00
	Sea Lion	1	0.09	5.21	5.54
	Sheep	12	0.39	4.65	4.81
	Squirrel	16	0.63	5.37	5.37
	Tiger	3	0.96	6.14	6.43
	Walrus	3	0.76	5.87	6.24
	Wart Hog	1	0.00	3.99	0
	Wolf	4	0.50	5.18	5.23
	Zebra	1	0.93	6.42	6.44
Reptile	Alligator	1	0.38	5.72	5.59
	Crocodile	6	0.23	5.21	5.36
	Lizard	17	0.58	5.04	5.15
	Snake	4	0.95	6.01	6.13
	Tortoise	1	0.13	6.20	6.11
	Turtle	10	0.88	5.74	5.86
Vertebrate	Fish	31	0.93	5.79	5.88

### Image and stimulus set analysis

The stimuli, raw data, as well as an excel spreadsheet containing specific details related to each stimulus are available on the Open Science Framework: http://doi.org/10.17605/OSF.IO/5DQU8. These details include the superordinate, basic and subordinate category of each image, the filename, image orientation, and size in pixels for each image, the number of ratings each image received, and their frequency across two corpora from the English Lexicon Project. In addition, we provide the strict and relaxed (see below) ratings of the following: the most frequent name associated with each image, the proportion of participants who provided the most frequent name, the means and standard deviations for name agreement and the quality ratings, alternative names provided in decreasing order, the proportion of responses fitting those alternative names, and the H-statistic of each image. In contrast to others (e.g.[1]) incorrect spellings were not included as correct. In some cases, there was no way to be certain whether participants misspelled the word or were not completing the task with full attention. 10 images had to be removed because the images could not be located in their original form, and thus permission could not be requested.

#### Accuracy ratings

Two types of accuracy were calculated. Strict accuracy ratings reflect the accuracy with which participants reported the basic category name for an exact match, and relaxed accuracy ratings reflect when a participant’s response contained the target word (e.g. if participants typed tabby cat this would be counted as correct in the relaxed criterion). Spelling errors or typos were counted as incorrect, and as such, these ratings are likely a slight underestimate of actual accuracy. Mean ratings by category both overall and for accurate responses only are presented in Table 1 while the ratings for each image (accurate only) can be found on our OSF page (http://doi.org/10.17605/OSF.IO/5DQU8).

#### Naming agreement

The overall H statistics, percent agreement ratings across all images for both strict and relaxed criteria are presented in Table 2. The H-statistic was calculated using the formula: $\text{H}=\sum_{i=1}^{k}p_{i}\text{log}2(1/p_{i})$, where k refers to the total number of names provided for that image, and *p*_*i*_ is the proportion of participants providing that specific name.

**Table 2:** Summary Statistics for all variables.

	Strict		Relaxed		
	H_s_	IA	H_r_	IA	Rating
M	1.09	0.79	.99	.81	5.6
SD	1.02	0.21	1.02	.21	.75
Median	0.73	0.89	.57	.92	5.84
Q1	0.31	0.66	.23	.69	5.22
Q3	1.7	0.95	1.51	.97	6.17
Range	0–4.8	0.13–1	0–5	0.1–1	2.54–6.67
Skew	2.23	0.29	2.77	.23	.54

H = H-statistic; IA = image agreement which represents the proportion of participants that agreed on the most common referent; Rating = overall rating of the images M = mean; SD = standard deviation; Q1 = 1^st^ quartile, Q3 = 3^rd^ quartile, Skew = (Q3-mdn)/(mdn-Q1). Strict = the spelling had to be exact and only contain the basic category (e.g. bear); Relaxed = the spelling had to be exact and contained at least the basic category (e.g. polar bear).

We excluded any incorrect spellings and, as such, the H-statistics are slightly higher than have been reported in other studies. The first set of statistics is based on a strict exact match (e.g. if the image was a bear and the participant typed polar bear, this was considered incorrect, see columns H-P). The second set of statistics used a slightly looser criterion such that, if participants used the target word in their response (e.g. tabby cat for cat), this was included as correct (see columns Q through W).

For both the strict and relaxed criteria, we also include the overall mean, standard deviation, median, range, 1^st^ and 3^rd^ quartiles (Q1 and Q3 respectively), and Skew (measured as (Q3-mdn)/(mdn-Q1) with values >1 indicating positive skew) to facilitate the selection of concepts at the center (or extremes) of the distribution (Table 2). The degree to which participants agreed on the identity of the images was approximately 80% across all images and ranged from 12 to 100%. As percentage agreement varies with complexity, this stimulus set provides a range of easy to difficult images from which researchers can select on the basis of their experimental questions. For the category of dog, the H-statistic was slightly higher than expected, as this category of animal is commonly encountered. This was related to the fact that participants often tried (correctly or incorrectly) to name the breed, or subordinate category of the animal (e.g. poodle), which does not contain the target word that represents the basic category (dog). This suggests that for this animal (and to a lesser extent the bird images), the subordinate category was more accessible than the basic category and provides researchers important information that can be used to select a subset of images that suit their needs (e.g. using only animals that are frequently referred to at the basic category level).

## Discussion

There are some limitations of the stimulus set and the norming procedure. First, although the maximum size of the images presented was 500*500 pixels, some of the images were smaller than this during the presentation. Images were all clearly visible, but the effect of size on agreement is unclear. Second, the animals were not limited in their orientation in that some of them were presented in top view, while others were presented in profile. The orientation of each image is also made available for the stimulus set, but the relationship between stimulus orientation and ratings is unknown.

The goal of developing this stimulus set was to provide researchers with a normed set of colored images of animals across broad superordinate, basic and subordinate categories that can be used in a variety of experimental settings. The versatility of this stimulus set and the fact that it was normed on untrained participants who were not asked to name a particular level (e.g. basic or subordinate) of image identity provides information regarding the natural manner in which naïve observers tend to respond. We hope that this will be a useful resource for researchers to answer their questions of interest.

## References

[1] Snodgrass JG, Vanderwart M. A standardized set of 260 pictures: norms for name agreement, image agreement, familiarity, and visual complexity. J Exp Psychol [Hum Learn]. 1980;6: 174–215. 10.1037//0278-7393.6.2.174 7373248

[2] Cycowicz YM, Friedman D, Rothstein M, Snodgrass JG. Picture naming by young children: norms for name agreement, familiarity, and visual complexity. J Exp Child Psychol. 1997;65: 171–237. doi: 10.1006/jecp.1996.2356 9169209 10.1006/jecp.1996.2356

[3] Wagemans J, De Winter J, Op de Beeck H, Ploeger A, Beckers T, Vanroose P. Identification of everyday objects on the basis of silhouette and outline versions. Perception. 2008;37: 207–244. doi: 10.1068/p5825 18456925 10.1068/p5825

[4] Dalrymple KA, Gomez J, Duchaine B. The Dartmouth Database of Children’s Faces: Acquisition and Validation of a New Face Stimulus Set. Urgesi C, editor. PLoS ONE. 2013;8: e79131 doi: 10.1371/journal.pone.0079131 24244434 10.1371/journal.pone.0079131 PMC3828408

[5] Tottenham N, Tanaka JW, Leon AC, McCarry T, Nurse M, Hare TA, et al The NimStim set of facial expressions: judgments from untrained research participants. Psychiatry Res. 2009;168: 242–249. doi: 10.1016/j.psychres.2008.05.006 19564050 10.1016/j.psychres.2008.05.006 PMC3474329

[6] Barbarotto R, Laiacona M, Macchi V, Capitani E. Picture reality decision, semantic categories and gender. Neuropsychologia. 2002;40: 1637–1653. doi: 10.1016/S0028-3932(02)00029-5 11992652 10.1016/s0028-3932(02)00029-5

[7] Bonin P, Peereman R, Malardier N, Méot A, Chalard M. A new set of 299 pictures for psycholinguistic studies: French norms for name agreement, image agreement, conceptual familiarity, visual complexity, image variability, age of acquisition, and naming latencies. Behav Res Methods Instrum Comput. 2003;35: 158–167. doi: 10.3758/BF03195507 12723790 10.3758/bf03195507

[8] Rossion B, Pourtois G. Revisiting Snodgrass and Vanderwart’s object pictorial set: the role of surface detail in basic-level object recognition. Perception. 2004;33: 217–236. doi: 10.1068/p5117 15109163 10.1068/p5117

[9] Victor TA, Furey ML, Fromm SJ, Ohman A, Drevets WC. Relationship between amygdala responses to masked faces and mood state and treatment in major depressive disorder. Arch Gen Psychiatry. 2010;67: 1128–1138. doi: 10.1001/archgenpsychiatry.2010.144 21041614 10.1001/archgenpsychiatry.2010.144 PMC3253452

[10] Waxman S, Gelman R. Preschoolers’ use of superordinate relations in classification and language. Cogn Dev. 1986;1: 139–156. doi: 10.1016/S0885-2014(86)80016-8

[11] Schendan HE, Kutas M. Neurophysiological evidence for transfer appropriate processing of memory: processing versus feature similarity. Psychon Bull Rev. 2007;14: 612–619. 17972722 10.3758/bf03196810

[12] Arnold NR, Bröder A, Bayen UJ. Empirical validation of the diffusion model for recognition memory and a comparison of parameter-estimation methods. Psychol Res. 2015;79: 882–898. doi: 10.1007/s00426-014-0608-y 25281426 10.1007/s00426-014-0608-y PMC4534506

[13] Snodgrass JG, Yuditsky T. Naming times for the Snodgrass and Vanderwart pictures. Behav Res Methods Instrum Comput. 1996;28: 516–536. doi: 10.3758/BF03200540 10.3758/bf03200741 10633980

[14] Russakovsky O, Deng J, Su H, Krause J, Satheesh S, Ma S, et al ImageNet Large Scale Visual Recognition Challenge. Int J Comput Vis. 2015;115: 211–252. doi: 10.1007/s11263-015-0816-y

[15] Hutchison D, Kanade T, Kittler J, Kleinberg JM, Mattern F, Mitchell JC, et al What Does Classifying More Than 10,000 Image Categories Tell Us? In: Daniilidis K, Maragos P, Paragios N, editors. Computer Vision—ECCV 2010. Berlin, Heidelberg: Springer Berlin Heidelberg; 2010 pp. 71–84. doi: 10.1007/978-3-642-15555-0_6

[16] Zhou B, Lapedriza A, Xiao J, Torralba A, Oliva A. Learning Deep Features for Scene Recognition using Places Database In: Ghahramani Z, Welling M, Cortes C, Lawrence ND, Weinberger KQ, editors. Advances in Neural Information Processing Systems 27. Curran Associates, Inc; 2014 pp. 487–495. http://papers.nips.cc/paper/5349-learning-deep-features-for-scene-recognition-using-places-database.pdf

[17] Fenson L, editor. MacArthur-Bates Communicative Development Inventories: user’s guide and technical manual. 2nd ed Baltimore, Md: Paul H. Brookes Pub. Co; 2007.

[18] Alario F-X, Ferrand L. A set of 400 pictures standardized for French: Norms for name agreement, image agreement, familiarity, visual complexity, image variability, and age of acquisition. Behav Res Methods Instrum Comput. 1999;31: 531–552. doi: 10.3758/BF03200732 10502875 10.3758/bf03200732

[19] Lachman R. Uncertainty effects on time to access the internal lexicon. J Exp Psychol. 1973;99: 199–208. doi: 10.1037/h0034633

[20] Morrison CM, Ellis AW, Quinlan PT. Age of acquisition, not word frequency, affects object naming, not object recognition. Mem Cognit. 1992;20: 705–714. doi: 10.3758/BF03202720 1435273 10.3758/bf03202720

[21] Barry C, Morrison CM, Ellis AW. Naming the Snodgrass and Vanderwart Pictures: Effects of Age of Acquisition, Frequency, and Name Agreement. Q J Exp Psychol Sect A. 1997;50: 560–585. doi: 10.1080/783663595

[22] Paivio A, Clark JM, Digdon N, Bons T. Referential processing: Reciprocity and correlates of naming and imaging. Mem Cognit. 1989;17: 163–174. doi: 10.3758/BF03197066 2927314 10.3758/bf03197066

[23] Balota DA, Yap MJ, Hutchison KA, Cortese MJ, Kessler B, Loftis B, et al The English Lexicon Project. Behav Res Methods. 2007;39: 445–459. doi: 10.3758/BF03193014 17958156 10.3758/bf03193014

[24] Dahan D, Magnuson JS, Tanenhaus MK. Time course of frequency effects in spoken-word recognition: evidence from eye movements. Cognit Psychol. 2001;42: 317–367. doi: 10.1006/cogp.2001.0750 11368527 10.1006/cogp.2001.0750

[25] Lachman R, Shaffer JP, Hennrikus D. Language and cognition: Effects of stimulus codability, name-word frequency, and age of acquisition on lexical reaction time. J Verbal Learn Verbal Behav. 1974;13: 613–625. doi: 10.1016/S0022-5371(74)80049-6

[26] Carroll JB, White MN. Word Frequency and Age of Acquisition as Determiners of Picture-Naming Latency. Q J Exp Psychol. 1973;25: 85–95. doi: 10.1080/14640747308400325

[27] Gilhooly KJ, Gilhooly ML. Age-of-acquisition effects in lexical and episodic memory tasks. Mem Cognit. 1979;7: 214–223. doi: 10.3758/BF03197541

[28] Kuperman V, Stadthagen-Gonzalez H, Brysbaert M. Age-of-acquisition ratings for 30,000 English words. Behav Res Methods. 2012;44: 978–990. doi: 10.3758/s13428-012-0210-4 22581493 10.3758/s13428-012-0210-4

[29] Wu YC, Lewis J, Staab J, Potentials B, Shao J, Holder BE, et al. Objective Visual Complexity as a Variable in Studies of Picture Naming. 1999.

[30] Mathworks. MATLAB The Language of Technical Computing. 2005.

[31] Brad Wyble, Gregory Wade, Michael Hess. Stream. Open Science Framework; 2018.

[32] Brainard DH. The Psychophysics Toolbox. Spat Vis. 1997;10: 433–436. 9176952

